# Adapting an in-person acceptance and commitment therapy–based psychological intervention for fear of cancer recurrence into a digital format: a real-world evaluation during the COVID-19 pandemic

**DOI:** 10.1007/s00520-025-09946-0

**Published:** 2025-09-27

**Authors:** Fiona Sinclair, David Gillanders, Christopher Hewitt, Natalie Rooney, Christine Bonathan, Lauren McAllister, Marta Correia, Lynsey Devlin, Kirsty Hendry

**Affiliations:** 1 NHS Greater Glasgow & Clyde, Glasgow, Scotland; 2 School of Health in Social Sciences, University of Edinburgh, Edinburgh, Scotland; 3 NHS Lothian, Edinburgh, Scotland; 4 Maggie’s Forth Valley, Larbert, Scotland; 5 Beatson Cancer Charity, Glasgow, Scotland; 6 School of Health & Wellbeing, University of Glasgow, Level 2 Clarice Pears Building, 90 Byres Road, Glasgow, G12 8TB Scotland

**Keywords:** Breast cancer, Fear of cancer recurrence, Acceptance and commitment therapy, Supportive care

## Abstract

**Purpose:**

Service evaluation comparing the effectiveness and acceptability of an in-person and a digitally delivered acceptance and commitment therapy (ACT)–based group programme for fear of cancer recurrence (FCR) for breast cancer survivors.

**Methods:**

The programme was designed and delivered as part of a real-world, supportive care intervention and adapted for digital delivery in response to COVID-19 pandemic restrictions. FCR, quality of life (QoL), psychological flexibility, and psychological distress measures were routinely collected pre-participation, post-participation, and 12 weeks following completion.

**Results:**

Ninety-seven in-person and 61 digital participants completed the group programme. Uptake was 30% and retention 89% for in-person. Uptake was 23% and retention 64% for online referrals.

Between group statistical analyses revealed non-significant differences between delivery modality on overall scores of FCR (*p* = 0.76), QoL (*p* = 0.06) and psychological distress (anxiety *p* = 0.16; depression *p* = 0.22). There was a significant difference in psychological flexibility scores (*p* = 0.04); digital participant scores were higher (mean = 83) than in-person participants (mean = 76.3). Within-group statistical analyses found a significant effect of time for all outcome measures, with a significant decrease in FCR and psychological distress and a significant increase in QoL and psychological flexibility (*p* < 0.001 for all measures). There were no statistically significant interaction effects between delivery modality and timepoint.

**Conclusion:**

In-person and online delivery of a real-world FCR group programme offered to breast-cancer survivors was found to be beneficial and comparable. These results support the potential benefits of a flexible approach to delivery modality of supportive care interventions. Further investigation is required to determine if these results are replicable within diverse populations.

## Introduction

Anxiety and worry relating to the recurrence of cancer is a normal response following curative treatment for cancer. Low levels can be beneficial and provide an adaptive function in ensuring ongoing engagement with healthcare follow-up, making positive lifestyle changes and attending to symptoms [1]. However, elevated levels of fear of cancer recurrence (FCR) can have a negative impact, leading to high levels of distress, overutilisation of healthcare services, persistent information seeking, and preoccupation with recurrence. FCR is acknowledged as a potentially serious, prevalent, and enduring condition and an unmet need of cancer survivors [2].

Psychological interventions can be effective in treating FCR, and contemporary cognitive behavioural therapy (CBT) interventions such as acceptance and commitment therapy (ACT) are found to be more beneficial than traditional CBT [3]. A recent systematic review and meta-analysis of 16 randomised controlled trials utilising ACT-based interventions concluded that such interventions are associated with a reduction in anxiety and depression while increasing psychological flexibility for people affected by cancer, when compared to control interventions [4].

Increasing psychological flexibility is the core aim of an ACT-based model and is defined as the ability to embrace the present moment, including challenging thoughts, feelings, and emotions. An ACT-based approach emphasises the need to understand what is truly important to an individual to enable them to live a values-based life alongside internal and external experiences [5].

ConquerFear, a partially ACT-based psychological intervention, provides insight into the efficacy and delivery adaptability of psychological intervention for FCR. ConquerFear was initially developed as a five-session, one-to-one in-person intervention in a randomised controlled trial (RCT) evaluation of 222 breast, colorectal, and melanoma cancer survivors. It demonstrated significant improvements in outcome measures of FCR, quality of life (QoL), distress, and anxiety [6]. Pilot [7] and RCT [8] evaluation of the ConquerFear intervention in a group setting demonstrated the ability to statistically significantly reduce FCR, persisting 6 months following group participation, for breast cancer survivors. The authors discuss the need for these promising results to be demonstrated within a real-life setting [8].

The intervention described within this service evaluation encompasses an ACT-based, psychological group programme for breast cancer survivors seeking support for FCR, the foundations of which were inspired by the ConquerFear intervention. This was initially developed by healthcare professionals as an in-person intervention, launched in 2017. Published service evaluation of this real-world, in-person group intervention shows statistically significant reductions in FCR and psychological distress alongside increased QoL and psychological flexibility, persisting at 12-week follow-up [9].

The COVID-19 pandemic caused a widespread negative psychological impact across society [10]. This shift towards virtual methods occurred in the absence of an evidence-based approach, due to the immediate requirement to reduce COVID-19 transmission. A systematic review comparing in-person and virtual care interventions from January 2015 to August 2020 found a lack of published research on the evaluation of clinical outcomes and supportive care in cancer [11].

To ensure ongoing support of people with FCR, the group programme was rapidly adapted for online delivery as a pragmatic response to pandemic-driven service adaptation. Here, we provide a real-world, descriptive evaluation of the effectiveness and acceptability of in-person and online delivery of an ACT-based group programme for FCR. Scores on measures of FCR, QoL, psychological distress, and psychological flexibility were compared between groups. Specific hypotheses are not defined due to the naturalistic and descriptive attributes of this evaluation.

## Materials and methods

### Group participants

The eligibility criteria for this group programme are as follows:

- Completion of curative, active treatment for a primary breast cancer
- No clinical evidence of disease
- Age 18 and over
- English fluency

Prescription of maintenance treatments such as endocrine therapy, Herceptin, and adjuvant bisphosphonates was not an exclusion to group participation.

Referrals to the group programme were received from NHS clinicians, charity organisations, and self-referral routes.

Referrals were deemed ineligible if they were in the process of receiving any form of primary treatment for cancer, had metastatic disease, or demonstrated evidence within their medical notes of complex psychological conditions, suicidal intent, or current alcohol/drug misuse.

### Service development and delivery

The group programme was originally developed by two clinical psychologists (CH, NR) and a specialist radiation therapist (FS) to be delivered as an NHS service provision [9]**.** The development of the in-person intervention was further informed by supervision from a clinical psychologist with ACT training and supervision expertise (DG). The programme was designed as an in-person intervention, taking place in hospital and community settings. The weekly sessions were designed to incorporate the six core ACT processes: acceptance, cognitive defusion, present moment awareness, self-as-context, values, and committed action [5, 9]**.** The in-person group programme launched in January 2017. Outcome measure data was routinely collected from all in-person groups running from inception to December 2019. Two psychologists were involved in group facilitation separately during this specified time (NR, CB) as well as a specialist radiation therapist (FS) who co-facilitated all sessions.

In response to the COVID-19 pandemic, the programme materials were modified by the group facilitators for online delivery. The materials were amended by the specialist radiation therapist who originally co-developed the intervention (FS) as well as a clinical psychologist (CB). The facilitators aimed to keep the programme as similar as possible to in-person delivery, with focus on the practicalities of achieving this, including utilising breakout rooms for smaller, non-facilitator group discussion, digital slideshows instead of flipcharts, and modification to experiential activities to accommodate a virtual format while maintaining fidelity to ACT principles. The first digital group was delivered in May 2020. The group programme was exclusively offered digitally via ‘Zoom’ between May 2020 and August 2022, and outcome measures were collected from all groups running during this period. Three psychologists were involved in group facilitation separately during this specified time (CB, LM, MC) as well as a specialist radiation therapist (FS) who co-facilitated all sessions.

For both delivery modalities, groups encompassed a maximum of 15 participants, taking place over 6 consecutive weeks, 2 h per week. All participants were invited to a follow-up session, which took place 12 weeks after completion.

See Table 1 for breakdown of weekly material delivery and difference between the delivery modalities.

**Table 1:** Weekly breakdown of content delivered, separated by delivery modality & differences between the delivery highlighted. Table abbreviated from publication Sinclair et al. [9] to include online group delivery

Session	In-person	Online	Differences
**1**	- Small group Ice breaker - Discuss/note on flipchart: i) Expectations of the group ii) Explanation of ACT and aim of group iii) Explore participant’s current coping styles and their impact Leaves on a stream mindfulness exercise	- Small group ice-breaker Discuss/with use of slides - Expectations of the group - Explanation of ACT and aim of group - Explore participant’s current coping styles and their impact Leaves on a stream mindfulness exercise	- Change to digital breakout room ice-breaker exercise - Digital slideshow used rather than paper flipchart
**2**	- Group discussion of the triggers of FCR - Educational group discussion on cancer recurrence, including signs and symptoms -Interactive exploration of the facts relating to lifestyle in small groups within the room -Three-minute breathing space mindfulness exercise	-Group discussion of the triggers of FCR - Educational group discussion on cancer recurrence, including signs and symptoms -Interactive exploration of the facts relating to lifestyle in small groups within a digital breakout room -Three-minute breathing space mindfulness exercise	-Digital slideshow used rather than paper flipchart -Interactive activity relating to facts done as a small group quiz in face-to-face setting was changed to a small group discussion in digital breakout rooms
**3**	- Group discussion: goals vs. values video by Russ Harris played - Value card exercise -Values bullseye exercise - Group discussion on the impact of our thoughts on our feelings and actions. Metaphors used to aid this discussion -Mindfulness of the hand exercise	- Group discussion: goals vs. values video by Russ Harris played - Value checklist shared and small group discussion -Values bullseye exercise - Group discussion on the impact of our thoughts on our feelings and actions. Metaphors used to aid this discussion -Mindfulness of the hand exercise	-Values card sorting exercise changed to breakout room discussion -Interactive bullseye changed from flipchart to a digital group discussion -Metaphors are discussed in person with no visual aid; slideshow used for digital
**4**	-Revisit bullseye exercise -Small group goal setting exercise - Larger group discussion on the impact of thoughts -Defusion tools given to help participants step back from tricky thoughts. Thanking your mind and passengers on the bus video from Russ Harris -Passengers on the bus - Mindfulness eating exercise	- Revisit bullseye exercise -Small group work to support each other with ideas on goals they can set - Group discussion on thoughts -Defusion tools to help participants step back from tricky thoughts - Thanking your mind and passengers on bus video from Russ Harris - Mindfulness eating exercise	- As session 3 with the bullseye - Digital breakout rooms -Digital discussions supported with relevant images on slides - Passengers on the bus acted in person by participants/digital animated video - Chocolate provided in-person for mindfulness exercise vs providing own for digital
**5**	- Revisit bullseye exercise - Bold move exercise—group - Exploration of barriers to living in line with values—small group - Impact of emotions discussion - Self-compassion discussion - Acceptance of emotions mindfulness exercise	- Revisit bullseye exercise -Bold move exercise—individual -Exploration of barriers to living in line with values—small group - Impact of emotions discussion - Self-compassion discussion -Acceptance of emotions mindfulness exercise	- Bullseye as session 3 - In-person bold move a group activity/on own digitally -Digital breakout rooms - Use of slideshow for metaphors, digitally
**6**	- Revisit bullseye exercise -Long-term values -Choice point tool -Course recap - Another kind of self mindfulness exercise	- Revisit bullseye exercise - Long-term values -Choice point tool - Course recap - Another kind of self mindfulness exercise	- Bullseye as session 3 - Digital breakout rooms - Use of slideshow for metaphors, digitally

### Data collection

Paper outcome measure questionnaires were sent by postal mail at three timepoints: pre-group participation, directly following the final session, and 12 weeks after group participation for both in-person and online modalities. Written explanation was provided alongside the outcome measures to explain their importance in monitoring the effectiveness of the group programme. Return of outcome measures was not enforced, and participants’ ability to attend the intervention was not dependent on their return.

#### Primary outcomes

Reduction in FCR was the primary aim of this intervention and was measured using the Fear of Cancer Recurrence Inventory-Short Form (FCSRI-SF) [12]**.**

#### Secondary outcomes

Secondary outcome measures included QoL measured by the Functional Assessment of Cancer Therapy for breast cancer (FACT-B) [13], psychological flexibility measured using the Comprehensive Assessment of Acceptance and Commitment Therapy Processes (CompACT) [14, 15], and psychological distress measured using the Patient Health Questionnaire (PHQ-9) [16] and the Generalised Anxiety Disorder Assessment (GAD-7) [17].

QoL (FACT-B) was assessed as a key outcome in supportive care in cancer [18]. Psychological flexibility (CompACT) was measured as this is the core process targeted by ACT interventions [14], offering insight into the effectiveness of treatment mechanisms. Measures of psychological distress (PHQ-9 and GAD-7) were selected due to their well-established relationship with FCR and broader survivorship wellbeing [12]. All measures were validated, brief, and non-redundant, supporting parsimony while enabling comprehensive evaluation of intervention impact.

Uptake and retention data was evaluated to determine if there was any difference between in-person and digital participation. Dropout rate was determined as those who failed to attend a minimum of four of the six group sessions.

### Statistical analysis

Demographic statistics were used to summarise participant demographics and study uptake/retention rates. Chi-square tests of independence were applied to compare demographics and acceptability measures of uptake and retention between group delivery modalities.

To assess outcome measure data (FCR, QoL, psychological flexibility, and psychological distress), we used a 2 × 3 mixed design ANOVA, with delivery modality as the between-subjects factor and time as the within-subjects factor.

Continuous outcome data were assessed for statistical assumptions prior to analysis. Normality was visualised and tested using Shapiro–Wilk tests. Assumption of sphericity for repeated-measures ANOVA was assessed using Mauchly’s test, and where this was violated, Greenhouse–Geisser correction was applied. Homogeneity of variance was tested using Levene’s Test. A *p* value of < 0.05 was used to infer statistical significance.

Mean scores and standard deviations with 95% confidence intervals were calculated for pairwise comparison across timepoints. Where significant effects of time were found, pairwise comparisons were conducted with Bonferroni correction for multiple comparisons. Partial eta squared values were reported to indicate effect size (small 0.01–0.05; medium 0.06–0.13; large ≥ 0.14). Analysis was carried out using v29 IBM SPSS Statistics**.**

### Ethical considerations

Ethical approval was not required as this intervention was routinely offered as an NHS provision of care and all data were recorded as standard. For purposes of this service evaluation, anonymised, routine data were analysed. Approval to access and analyse this anonymised data under ownership of the NHS health board was obtained from the Caldicott Guardian.

### Funding

The funding for the group programmes running between January 2017 and August 2022, and encompassing the data analysed within this service evaluation, was provided by Breast Cancer 2000 (SC030630), the Beatson Cancer Charity (SC044442), and the National Lottery Community Fund (Charity Number 1010518).

## Results

### Descriptive statistics

A total of 772 breast cancer survivors were referred to the 6-week FCR group programme between January 2017 and July 2022. This encompassed 361 referrals (13.9 per month) for the in-person programme between January 2017 and December 2019 and 411 referrals (13.7 per month) for the online programme between January 2020 and July 2022.

Group participants were all female and had completed curative treatment for breast cancer; mean age was 52 years (in-person delivery) and 49 years (digital delivery). The mean age of those who dropped out was 51 years (in-person) and 59 years (digital). The majority of participants who completed a group had completed treatment within 6 months: 68/97 (70%) in-person participants and 36/61 (59%) online participants. Descriptive statistics are detailed within Table 2**.** Chi-square analyses were conducted to examine differences in demographic characteristics, and no statistically significant differences in age, SIMD distribution, or ethnicity were found.

**Table 2:** Baseline routinely collected demographic information comparing in-person and online participants. Table adapted from publication Sinclair et al. to include online group participants data for comparison

	**Category**	**In-person participants n (%)**	**Online participants** **n (%)**
**Age range**	< 40	11 (11)	10 (16)
	40–50	30 (31)	22 (36)
	51–60	39 (40)	18 (30)
	> 61	17 (18)	11 (18)
**Scottish index of mutiple deprivation**	1	12 (12)	10 (16)
	2	25 (26)	10 (16)
	3	15 (15)	9 (15)
	4	19 (20)	15 (25)
	5	26 (27)	17 (28)
**Ethnicity**	White British	95 (97)	58 (95)
	White Polish	2 (3)	1 (2)
	Asian Indian	0 (0)	2 (3)
**Total sample**		97 (100)	61 (100)

Chi-square analyses were conducted to examine whether group delivery modality (in-person vs online) was associated with significant differences in uptake, retention, 12-week follow-up session attendance, and outcome measure return.

There was an uptake rate of 30% (*n* = 109/361) for patients offered an in-person group and 23% (*n* = 96/411) for patients offered an online group. Chi-square analyses indicated a significant difference in uptake between delivery modality; *χ*^2^(1) = 4.82, *p* = 0.028, with higher uptake observed in the in-person group programme.

The group programme retention rate was calculated based on completion of at least four out of six sessions. Eighty-nine per cent (*n* = 97/109) participants completed the in-person programme, and 64% (*n* = 61/96) completed the digital programme. Chi-square analyses indicated a statistically significant difference in retention between delivery modality: χ^2^(1) = 13.56, *p* < 0.001, with higher retention in the in-person group programme.

Of those who started an in-person group programme, 87% (84/109) attended the 12-week follow-up, compared to 74% (71/96) of those who started an online group. Chi-square analyses indicated this difference to be statistically significant; χ^2^(1) = 4.36, *p* = 0.037, indicating higher 12-week follow-up attendance for in-person group programme participants.

Of those who completed, 92% (*n* = 84/91) of in-person participants returned outcome measures at all three time points, compared to 61% (*n* = 37/61) of online participants. Chi-square analyses indicated this difference to be statistically significant; *χ*^2^(1) = 20.55, *p* < 0.001, with in-person participants more likely to return outcome measures.

**Fig. 1:**
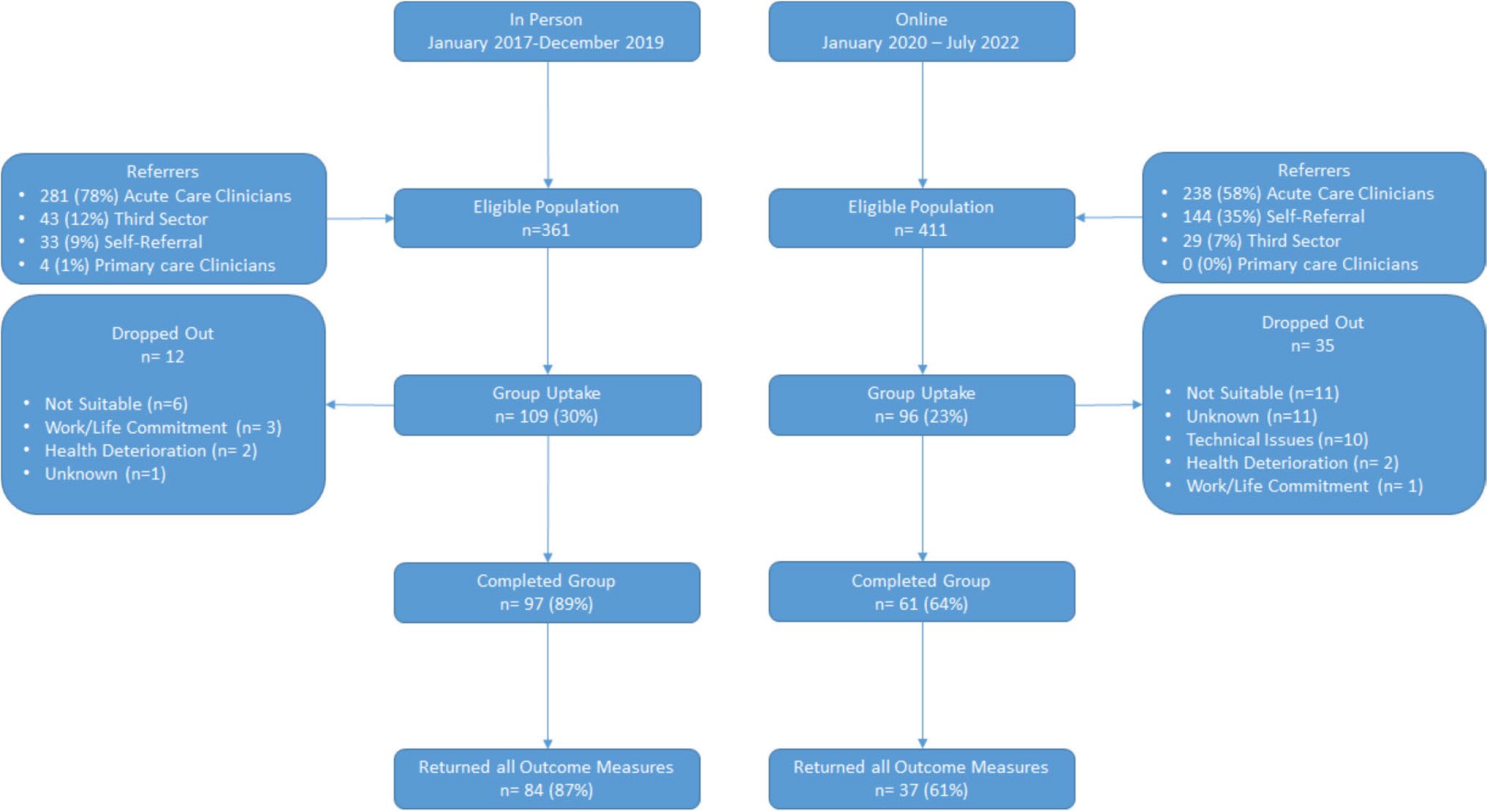
Flow diagram illustrating uptake, retention, drop-out, and outcome measure return for in-person and online group delivery

Figure 1 details the breakdown of source of referrals, drop-out reasons, uptake, and retention

### Primary outcomes

A 2 × 3 mixed ANOVA was conducted for all outcome measures.

Levene’s statistic supported the assumption of homogeneity of variance. Mauchly’s test of sphericity for the within groups data indicated that the assumption of sphericity has been violated, *X*^2^(2) = 0.93, *p* = 0.02. Greenhouse–Geisser correction was applied to within-subjects’ analyses.

There was no main effect of administration type, *F*(1,119) = 0.1, *p* = 0.76, *η*_*p*_^2^ = 0.001, indicating FCR scores were similar for in-person and online group participants, regardless of time.

There was found to be a significant main effect of time, *F*(1.9, 222.9) = 78.3, *p* < 0.001, *η*_*p*_^2^ = 0.40. Pairwise comparisons revealed a significant increase in scores across timepoints, at all combinations (T1 to T2, T1, to T3 *p* < 0.001 and T2 toT3 *p* < 0.01).

Analyses revealed a non-significant interaction effect, *F*(1.9, 222.9) = 0.60, *p* = 0.52, *η*_*p*_^2^ = 0.01, showing that the two modes of delivery changed at similar rates across time.

### Secondary outcomes

Levene’s statistic revealed no significant difference in distributions across groups for measures of QoL, psychological flexibility, depression, or anxiety, upholding the assumption of homogeneity of variance for all secondary outcome measures.

Machly’s test of sphericity revealed the assumption of sphericity was not violated for the measure of QoL (*X*^2^(2) = 0.97, *p* = 0.17). Sphericity was found to be violated for psychological flexibility (*X*^2^(2) = 0.86, *p* < 0.001), depression (*X*^2^(2) = 0.80, *p* < 0.001), and anxiety (*X*^2^(2) = 0.93, *p* = 0.02). Therefore, Greenhouse–Geisser correction was applied to within-subjects’ analyses for measures of psychological flexibility and psychological distress.

Analysis of the FACT-B data as a measure of QoL revealed a non-significant main effect of administration type, *F*(1,119) = 3.5, *p* = 0.06, *η*_*p*_^2^ = 0.03, indicating scores were similar for in-person and online group participants, regardless of time.

There was found to be a significant main effect of time, *F*(2, 238) = 100.4, *p* < 0.001, *η*_*p*_^2^ = 0.46. Pairwise comparisons revealed a significant increase in mean scores across time points, at all combinations (*p* < 0.001). There was found to be a non-significant interaction effect *F*(2,238) = 1.9, *p* = 0.15, *η*_*p*_^2^ = 0.02, showing that the two methods of administration did not differ in their improvement.

Analysis of the CompACT data as a measure of psychological flexibility revealed a significant main effect of administration type *F*(1,119) = 4.2, *p* = 0.04, *η*_*p*_^2^ = 0.03, with online participants having higher flexibility scores on average (mean = 83.0; SE = 1.8), compared to in-person participants (mean = 76.3, SE = 2.7).

There was found to be a significant main effect of time, *F*(1.8, 209.0) = 82.2, *p* < 0.001, *η*_*p*_^2^ = 0.41. Pairwise comparisons revealed a significant increase in mean scores at all timepoint combinations (T1 to T2, T1 to T3 *p* < 0.001 and T2 to T3 *p* < 0.01).

Analysis revealed a non-significant interaction effect *F*(1.8, 209.0) = 335.8, *p* = 0.11, *η*_*p*_^2^ = 0.02, further supporting the finding that the groups experienced similar improvements, even though they differed in level of psychological flexibility.

Analysis of the PHQ-9 data as a measure of anxiety revealed there was no significant main effect of administration type, *F*(1,119) = 2.0, *p* = 0.16, *η*_*p*_^2^ = 0.02, indicating similar scores for in-person and online group participants.

There was found to be a significant main effect of time, *F*(1.9, 223.4) = 53.2, *p* < 0.001, η_p_^2^ = 0.31. Pairwise comparisons revealed a significant decrease in mean scores between T1 and T2 (*p* < 0.001), T1 and T3 (*p* < 0.001), but not T2 to T3 (*p = *0.09).

There was found to be a non-significant interaction effect *F*(1.9, 223.4) = 19.0, *p* = 0.34, *η*_*p*_^2^ = 0.01, showing equivalence of improvements between the delivery modes.

No statistically significant main effect of administration type was found for anxiety, *F*(1,119) = 1.54, *p* = 0.22, *η*_*p*_^2^ = 0.13, indicating scores were similar for in-person and online group participants, regardless of time.

There was found to be a significant main effect of time, *F*(1.7, 197.9) = 47.4, *p* < 0.001, *η*_*p*_^2^ = 0.29. Pairwise comparisons revealed a significant decrease in mean scores between T1 and T2 (*p* < 0.001) and T1 to T3 (*p* < 0.001), but not T2 to T3 (*p* = 0.06).

Consistent with other variables, there was no interaction effect *F*(1.7, 197.9) = 0.05, *p* = 0.96, *η*_*p*_^2^ < 0.001.

Table 3 shows the details mean scores and between- and within-subject results for all outcome measures.

**Table 3:** Comparison of mean scores between administration types and results of 2 × 3 mixed ANOVA, evaluating between group variable of administration type and within group variable of time point

**Outcome measure**		**T1**	**T2**	**T3**	Administration *F*_(1,119)_	*η*_*p*_^2^	Time point *F*_(2,238)_	*η*_*p*_^2^	Time point × administration *F*_(2,238)_	*η*_*p*_^2^
		**Mean (SD)**	**Mean (SD)**	**Mean (SD)**						
**FCR (FCRI-SF)**	**In-person**	25.2^a^ (4.7)	21.2^b^ (5.4)	19.5^c^ (6.2)	0.1	0.001	78.3^† ***^	0.40	0.6^†^	0.01
	**Online**	25.4^a^ (5.3)	20.3^b^ (6.3)	19.3^b^ (6.9)						
**QoL (FACT_B)**	**In-person**	62.4^a^ (15.7)	71.7^b^ (18.1)	75.9^c^ (17.5)	3.5	0.03	100.4 ***	0.46	1.9	0.02
	**Online**	65.5^a^ (16.1)	78.5^b^ (16.4)	83.2^c^ (16.6)						
**Psychological Flexibility** **(CompACT)**	**In-person**	66.7^a^ (14.3)	79.0^b^ (18.6)	83.3^c^ (19.4)	4.2*	0.03	82.2^†^ ***	0.41	2.3^†^	0.02
	**Online**	69.5^a^ (22.0)	87.9^b^ (22.4)	91.6^b^ (20.4)						
**Depression (PHQ-9)**	**In-person**	10.2^a^ (5.4)	7.5^b^ (5.6)	6.5^b^ (6.1)	2.0	0.16	53.2^†^ ***	0.31	19.0^†^	0.01
	**Online**	9.4^a^ (6.4)	5.5^b^ (4.5)	5.2^b^ (5.1)						
**Anxiety (GAD-7)**	**In-person**	10.0^a^ (5.5)	6.8^b^ (5.4)	6.1^b^ (5.8)	1.54	0.13	47.4^†^ ***	0.29	0.5^†^	0.001
	**Online**	8.9^a^ (5.8)	5.5^b^ (4.8)	4.9^b^ (5.1)						

Means with different superscripts (a, b, c) are significantly different for within participant comparisons at the level of *p* < .05

^*^p < 0.05, **p < 0.01, ***p < 0.001

^†^ indicates altered degrees of freedom with Greenhouse–Geisser correction

Effect size for partial eta squared (*η*_*p*_^2^) can be described as following: small = 0.00–0.05, medium = 0.06–0.13, large = ≥ 0.14

## Discussion

### Summary

This service evaluation compared in-person and online delivery of a real-world 6-week supportive care intervention for FCR after breast cancer. The intervention was found to benefit participants across all outcomes of both delivery modalities, reducing FCR and psychological distress, while increasing QoL and psychological flexibility*.* Statistical analysis revealed that in-person and online group participants’ outcome scores on measures of FCR, QoL, and psychological distress were comparable for in-person and online delivery. Psychological flexibility scores, overall, were shown to be higher for online group participants; results were statistically significant and with a small effect size. There were found to be no statistically significant interaction effects between group delivery method and time on any measure.

We recognise the importance of interpreting if these findings translate to minimal clinically important difference (MCID). While no universally accepted MCID exists for the FCRI-SF, studies have suggested that reductions of 4–5 points are considered clinically meaningful [6, 19]. Our within-group reduction from baseline to 12-week follow-up exceeded this threshold for both delivery modalities. Furthermore, research suggests that a reduction of 7–8 points in QoL score using the FACT-B indicates MCID [13]. The observed mean improvements presented within this evaluation exceeded this for both groups.

We found the relatively rapid adaptation to online delivery to be well received, with similar numbers of referrals per month across the evaluated period. Clinician referrals reduced for the online group programme and self-referrals increased, as may be expected following pandemic restriction onset. However, the in-person retention rate was significantly higher (89%) and comparable to other published work on FCR intervention, finding a retention rate of 94% [19]. We found a significantly lower retention rate of digital groups (64%), despite focussed attention on comparable delivery materials. However, our digital group retention rate was comparably higher than the digital iConquerFear intervention of 36% [20], an intervention which utilises user-led delivery rather than facilitator-led. Within real-world healthcare interventions, there is a lack of empirical evidence to compare these findings. However, evaluation within education settings suggests dropout for online courses compared to in-person is 15–20% higher [21]. It was also found that in-person participants were more likely to attend the 12-week follow-up session and more likely to return outcome measures.

### Impact of COVID-19 pandemic

Evidence emerging following the end of pandemic restrictions revealed that psychological support continued to be important for many affected by cancer [22]. Within our evaluation, the in-person outcome measure data precedes the COVID-19 pandemic, whereas the online group data collection started soon after onset. It is reasonable to conclude that outcome scores may be impacted. However, our results revealed no main effect of delivery modality; therefore, suggesting outcome scores were not impacted by the pandemic within this cohort. A scoping review of the potential impact of the pandemic on cancer survivors (26% breast), based on a total of 4831 people, revealed a statistically significant increase in distress and FCR compared to similar patient populations pre-pandemic [23]**.** However, our evaluation revealed no comparable between-group difference on FCR, anxiety, or depression scores. This disparity in findings may be due to several factors, including the differing impact of the pandemic on health and care services across geographic regions [24], the patients within our evaluation being breast cancer specific and, overall, not being representative of wider populations. Systematic review evidence pooling studies encompassing 27,356 people with cancer suggests it is too early to know the psychological impact of the COVID-19 pandemic on people affected by cancer compared to general populations [25]**.**

Following the gradual return to in-person healthcare service provisions, many services have continued to offer hybrid or online-only support, presenting the opportunity for comparison of delivery modality on patient outcomes. A recent cancer support service audit compared patient and therapist experience of in-person versus online delivery. Qualitative data revealed patients felt understood, respected, and confident in their ability to engage effectively with online support. Patient feedback highlighted the benefits of accessibility and reduced risk of infection. However, quantitative data revealed 87% of patients and 100% of therapists preferred in-person support [26].

The results presented within our service evaluation contribute to the pool of data emerging following the end of pandemic restrictions. This has allowed for the comparison of real-world, supportive care interventions delivered in-person and online. As such, we have demonstrated the possibility of an acceptable FCR group intervention deliverable via in-person and online methods, with positive outcomes for breast-cancer survivors. However, uptake and retention were both significantly lower in the online group programme compared to in-person delivery. It remains unclear whether this reduced engagement is attributable to the acute effects of the COVID-19 pandemic or reflects a more persistent challenge associated with digital intervention delivery. A recent scoping review identified 28 web-based interventions for FCR and reported mixed evidence of effectiveness, with common limitations including insufficient long-term follow-up and poor reporting of participant adherence and completion rates [27]**.** The findings of our evaluation contribute real-world evidence suggesting lower engagement with online group-based interventions. Further research is needed to determine whether these trends persist beyond the pandemic context and to explore strategies for improving uptake and retention in digital FCR support programmes.

### Strengths

Our service evaluation demonstrates that a real-world supportive care intervention for breast cancer survivors improved FCR, QoL, psychological flexibility, and psychological distress outcomes following completion of a 6-week group programme. Furthermore, the intervention was able to be quickly adapted for online delivery and ensured continued supportive care during the pandemic when many supportive interventions for people affected by cancer were reduced or withdrawn [28]. We demonstrate the possibility of delivering an intervention for FCR both online and in person while adding to a limited pool of data on the experience of breast cancer patients during the global pandemic.

### Limitations

The results from our service evaluation are limited by the population, which consisted of breast cancer survivors. Furthermore, results are not generalisable outside the geographical region where this intervention was offered as part of routine healthcare intervention. The in-person and online delivery of groups were not delivered in parallel, limiting any conclusions drawn regarding comparability, and group participants did not have a choice of modality. Participants were not randomised to delivery format and did not have a choice in modality, which may have affected motivation, expectations, or accessibility. Finally, while outcome measures demonstrated statistically significant improvements across both formats, clinically meaningful change thresholds and qualitative participant experiences were not assessed, limiting our understanding of the personal relevance and sustainability of observed effects.

### Future directions

There are currently no UK clinical guidelines for cancer survivors living with FCR, despite recognition that this is an unmet need [29] and evidence indicating the long-term persistence of FCR within breast cancer survivors [30, 31]. Recent clinical guidelines in Canada have been published, developed by a multidisciplinary panel including patient representation, recommending a systematic, integrated approach to identification and treatment of FCR in cancer care settings [32]. These guidelines advocate early detection through screening at routine follow-up and appropriate triage to relevant intervention separated into low (education/brief intervention), medium (self-management or group intervention), and high FCR (specialist psychological care) need.

Rigorous research methodology would be of benefit to inform clinical guidelines, including the evaluation of optimal delivery modality as well as feasibility and effectiveness for other cancer types.

It would be of interest to assess whether patients having the choice of delivery modality would be beneficial.

Future evaluations should consider integrating routine assessment of digital readiness, comorbidities, and health literacy, alongside mixed-methods data collection, to explore barriers and facilitators of engagement more fully.

Further research is needed to evaluate the health economics of online versus in-person delivery.

## Conclusion

The results presented add to the limited but growing evidence of the efficacy of ACT for the treatment of FCR in a real-world, healthcare setting, over an extended period. Furthermore, we demonstrate the possibility of amending an in-person ACT intervention for online delivery, with encouraging results. This service evaluation demonstrates that a 6-week, ACT-based psychological intervention for FCR shows promise in improving a range of outcomes for breast cancer survivors, whether delivered in-person or online. This small-scale evaluation was based on mixed data collected routinely as part of a real-world supportive care service evaluation. Due to the nature of this service evaluation, the results are not generalisable to other settings or patient populations but provide justification for further investigation using rigorous research methodology. Overall, this evaluation adds to the growing body of evidence indicating the ability and need to effectively address FCR in breast cancer survivors through structured, psychological, group-based intervention.

## Data Availability

No datasets were generated or analysed during the current study.
